# Assessment of municipal solid waste from households in Khulna city of Bangladesh

**DOI:** 10.1016/j.heliyon.2023.e22446

**Published:** 2023-11-28

**Authors:** A.A. Noman, Islam M. Rafizul, S.M. Moniruzzaman, E. Kraft, S. Berner

**Affiliations:** a Department of Civil Engineering, Khulna University of Engineering & Technology (KUET), Khulna, 9203, Bangladesh; b Institute of Disaster Management, Khulna University of Engineering & Technology (KUET), Khulna, 9203, Bangladesh; c Faculty of Civil Engineering, Bauhaus-Universität Weimar (BUW), Germany

**Keywords:** Solid waste, Household, Characterization, Quantification and socioeconomic

## Abstract

Waste management is a major concern for both developed and developing countries, with a particular focus on household waste because it makes up a significant proportion of municipal waste. The aim of this study is to assess the state of solid waste management practice in Khulna, as well as to characterize and quantify municipal solid waste as a step toward effective management. To collect information on the existing waste management methods, structured questionnaires were used to conduct surveys of household residents. In this study, whole wards (31 wards) of Khulna City Corporation (KCC) were clustered in 9 groups and selected one ward from each group. To analyze household waste, 75 households from each ward were selected and collected waste for 7 days. The selected household was categorized into five different socioeconomic strata such as low-income, lower-middle-income, middle-income, higher-middle-income and higher-income families. Besides, the assessment was carried out on the production and characterization of household waste that was produced in KCC. The waste samples were quantified, separated and characterized in the laboratory. Results reveal that biodegradable waste is the most prominent type and its percentage is about 81 %. The amount of waste production is positively correlated with income level. The waste generation rate of households for high-income families was 0.652 kg/cap/day while this rate got almost half for a low-income family and its value is o.312 kg/cap/day. Source separation of waste plays a vital role to reduce plastic leakage to the SDP. The result shows the proportion of plastic in mixed waste and source-separated waste after sorting by the waste collector was 4.04 % and 2.99 %, respectively. Survey results show that 42.96 % of respondents think that the source-separated waste should be collected during the period of 12pm to 02pm. A proposed management process was developed for household waste based on the output of this study.

## Abbreviations

CBOs: Community-Based organizations
KCC: Khulna City Corporation
MSW: Municipal Solid Waste
NGOs: Non-Government Organizations
SD: Standard Deviation
SDG: Sustainable Development Goals
SDP: Secondary disposal Point
SPSS: Statistical Package for Social Sciences
SWM: Solid waste management
WGR: Waste generation rate

## 1 Introduction

The overall system for managing and regulating Municipal Solid Waste (MSW) is greatly influenced by the amount of waste generated at sources on a daily basis [1]^.^ The generation of MSW in developing-country cities has increased many times over the last few decades. Most of the countries which are developing have a remarkable rate of MSW production [2]. So, MSW has become a major issue in recent years, as waste generation has increased dramatically as a result of rapid industrialization and urbanization population growth, and improved living conditions. A developing country like Bangladesh, generate a large amount of domestic waste such as biodegradable waste plastics waste, paper and cardboard waste, textiles & wood waste, etc. through daily food, clothing, and other consumption Bangladesh is rapidly urbanizing, with massive internal rural-urban migration, leading to a rise in the population of cities. As a result, major cities in Bangladesh face urban environmental threats such as unorganized loads of solid waste, inadequate water supply, and sanitation [3].

A significant portion of municipal solid waste is generated from households including percentages of 55–80 while 10–30 % are generated by commercial areas or markets and the other percentage is generated by industries, streets, institutions, and others [4,5]. In Khulna city, the total amount of solid waste generation is found as 1000000 kg d-1 [6]. The Khulna city corporation (KCC) authority is responsible for waste management in this city [7]In Khulna city, municipal waste management is carried out by the conservancy section under the supervision of Khulna City Corporation's Mayor. Municipal solid waste is deposited on the roadside, community bin, container points or secondary disposal points by inhabitants or Community Based organizations (CBOs) and Non-Government Organizations (NGOs) via door-to-door waste collection. Generally, KCC employees do not collect municipal waste from sources such as households, streets, institutions markets, etc. They collect wastes from community bins or secondary disposal points and transport them to Rajbandh which is the final disposal site that is 10 km west of the city headquarters [8].

The waste generation in Khulna is rising proportionately with the increase in population, presenting a serious threat to solid waste management and disposal. Ethically, proper solid waste management system is the most essential task of the Khulna City Corporation (KCC) for keeping the city clean, healthy and hygienic. Before developing and implementing a sustainable solid waste management system for a specific location it is very essential to determine the waste generation rates and categorize waste composition. Furthermore, household solid waste should be quantified, analyzed and characterized in order to establish a waste collection system and treatment scheme for urban residential areas [9]. The research on household solid waste will provide valuable insights into the quantity and composition of municipal solid waste generated by households in Khulna City. This information is crucial for waste management planning and infrastructure development. It enables authorities to design appropriate waste management systems, including collection, transportation, and disposal strategies, based on the specific characteristics of the waste generated [10]. On the other hand, effective waste management is essential for minimizing the environmental impact of solid waste. Understanding the composition of municipal solid waste allows researchers to assess the potential environmental hazards associated with different waste components. It helps in identifying waste streams that require special treatment, such as hazardous materials or non-biodegradable waste, to prevent pollution of soil, water, and air [11]. Municipal solid waste often contains valuable resources that can be recovered and recycled. Research on waste composition enables the identification of recyclable materials, such as paper, plastic, glass, and metals, present in household waste streams. This knowledge can guide efforts to establish efficient recycling programs, reduce resource depletion, and promote a circular economy [4]. Research findings on municipal solid waste assessment provide scientific evidence for policymakers, local authorities, and relevant stakeholders to make informed decisions. This data can support the formulation of waste management policies, regulations, and guidelines at the municipal and national levels. It facilitates the allocation of resources, funding, and investments in waste management infrastructure and encourages the implementation of sustainable waste management practices [12].

Source separation of waste is essential for effective waste management and promoting sustainable practices. Source separation helps minimize the environmental impact of waste. By separating recyclables, organics, and non-recyclables at the source, valuable resources can be recovered and diverted from landfill or incineration. Recycling reduces the extraction of raw materials, conserves energy, and reduces greenhouse gas emissions. Separating organics allows for composting or anaerobic digestion, turning them into valuable soil amendments or biogas for energy generation [13]. Source separation helps streamline waste management processes. Separated waste streams can be collected, transported, and processed more efficiently and cost-effectively. Recycling facilities can focus on specific waste streams, optimizing their operations and increasing recycling rates. Separating organics reduces the volume and weight of mixed waste, reducing transportation costs and extending the lifespan of landfill sites [14]. Proper source separation of waste contributes to improved health and sanitation conditions. Separating organics from other waste streams reduces odors and the attraction of pests and rodents. It also minimizes the generation of leachate, a harmful liquid produced by decomposing waste, which can contaminate groundwater sources. Effective waste management practices reduce the risk of diseases and ensure a cleaner living environment [15]. Researcher Villa [16] conducted a study on the results of a collaboration between local authorities and researchers are presented and discussed that are the assessment of waste generation in the city of Quelimane (Mozambique), integrating existing and feld-collected data and the design of a small-scale center for plastic sorting to complement the SWM system of the city. Researcher Pandey [17] summarized currently reported techniques and approaches for the removal of plastic waste. Many techniques, such as adsorption, coagulation, photocatalysis, and microbial degradation, and approaches like reduction, reuse and recycling are potentially in trend and difer from each other in their efciency and interaction mechanism.

A few studies on the quantity and characteristics of household waste in Khulna City have been conducted for developing an effective decision toward a municipal solid waste management system to fulfill the Sustainable Development Goals (SDG). Researchers Alamgir and Ahsan [18]conducted a study on physical composition and daily waste generation which were determined by collecting municipal solid waste samples from various sources such as residential, commercial, institutional, and open areas in six major cities in Bangladesh, namely Dhaka, Khulna, Chittagong, Barisal, Sylhet and Rajshahi. Riyad [19]conducted a study and the goal of the study was to make a step forward in the systematic study of commercial solid waste management which will lead to the quantification of the amount of waste generated in the residential and commercial areas, the determination of its composition, and the quantification of the different composition characteristics of commercial solid waste. Islam [20] focused on the overview system of current waste management in Khulna city with regard to the quantity of managed municipal solid waste, daily driven distance, and the fuel consumption of collection and transportation trucks. Moniruzzaman [21] conducted a study on the scenario of the solid waste recycling practices of Khulna city, the quantity of recycled waste that impacts the economy. The researcher also proposed a model to evaluate the possibility of organizing the unorganized waste recycling practices that increase the efficiency of the present recycling process. Hossain [22] studied the generation, characteristics, and disposal system of municipal solid waste in Khulna city. The researcher conducted a survey to households to gather their opinion and information about municipal waste management in Khulna city. There is a very few research that has been conducted in Khulna City that shows the actual amount of waste generation by different socioeconomic groups.

Accurate data on the quantity and composition of municipal solid waste generated in Khulna City is essential for effective waste management planning. However, a lack of comprehensive studies or up-to-date data that provide detailed information on the types and amounts of waste generated by households. There is very few studies were found in the literature on household waste generation rate on income level. A lack of detailed data on the current status of recycling and resource recovery initiatives in Khulna City. Information on the recycling rates, types of materials recycled, and the functioning of recycling facilities can help identify gaps and opportunities for improvement in the recycling sector. On the other hand, data on the methods and facilities used for waste disposal in Khulna City may be incomplete. Data on the extent to which households in Khulna City practice source separation of waste is often limited. This includes information on the percentage of households that engage in source separation, the types of waste streams they separate, and the effectiveness of their separation practices. Obtaining accurate data on household-level source separation is crucial for understanding the current status and identifying areas for improvement.

The aim of this study is to assess the state of solid waste management (SWM) practice in Khulna, as well as to characterize and quantify municipal solid waste as a step toward effective management. Another goal was to assess of household income level influences the generation and composition of municipal waste, as well as the source separation of waste that plays a vital role to reduce plastic leakage to the secondary disposal points. Finally, a general physical model was proposed in consultation with the relevant stakeholders for its long-term sustainability.

## 2 Methodology

In order to obtain a representative waste sample for Khulna city, this study followed a stratified sampling approach. Two relevant subgroups or strata were identified: wards and income-groups. The following sections describe the selection process for these wards and households, the sampling based on income-level, waste analysis and finally the extrapolation of results to the whole study area.

### 2.1 Study area

Khulna is the third largest city in Bangladesh with an estimated population of 1.3 million [23]. It is situated in the southwest of the nation (between 21.38′N and 23.1 N Latitude and 88.58 E longitude) along the banks of the rivers Rupsha and Bhairab, approximately 12 ft above mean sea level. The city area is under the authority and management of Khulna City Cooperation (KCC). The cooperation comprises a total area of 14.30 square miles and is subdivided into 31 wards. The distribution of households based on wealth quantiles in Khulna is about 15.5% wealthiest and 23.6% poor. The literacy rate in KCC is 73.6 % [24]. Like many cities in Bangladesh, Khulna is experiencing rapid urbanization, resulting in increased waste generation and changing consumption patterns. In Khulna city, the total amount of solid waste generation is found as 1000 tons per day with a majority of food and vegetable wastes and the residential areas are the prime source of production [6]. Approximately half of the household-generated garbage is appropriately disposed of at designated dumping sites, while the larger portion remains uncollected and unregulated. In Khulna, the management of solid waste (SWM) falls under the purview of local administrations. The Chief Conservancy Officer (CCO) oversees this responsibility, operating under the direct supervision of the city corporation's Mayor. The primary and crucial duty of these officials is to ensure the proper handling and disposal of waste to enhance the overall cleanliness and hygiene of the city [18]. There are approaches that are seen to manage municipal waste in Khulna City. Firstly, At home, waste is produced and typically stored until a sufficient quantity has accumulated. The households that generate the waste are accountable for transporting it to the nearest roadside bin, which is provided by the city corporation. Subsequently, the city corporation takes charge of transferring this waste from the roadside bins to the ultimate disposal site. Secondly, the waste produced in households is stored and collected on a daily basis by a primary collector working for the NGO/CBO. This collector then transports the waste to nearby transfer points, typically using a rickshaw van. Afterward, the waste is gathered from the transfer points and transported to the final disposal site using a large truck. This phase of collection is referred to as secondary collection and is the responsibility of the city corporation [25]. There are approximately 16 NGOs and 2 CBOs are working KCC of solid waste management, whose prime duty is to collect waste from households (KCC 2023).

**Fig. 1:**
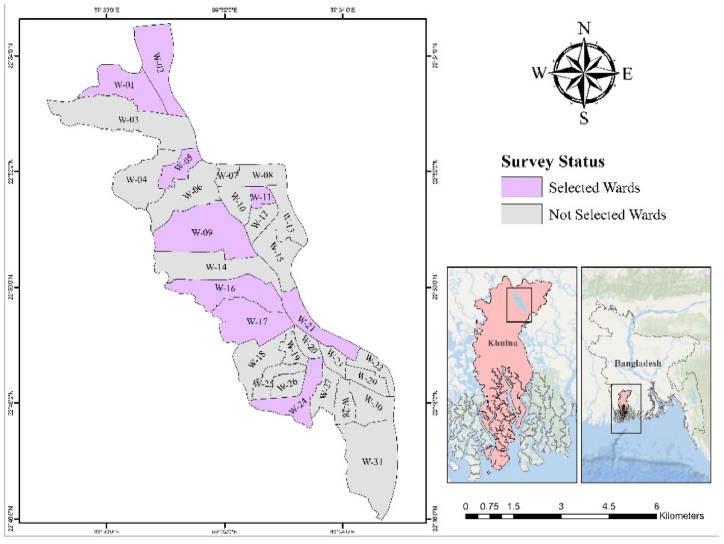
Map of KCC showing different selective wards for this study.

In this study, 9 wards (1, 2, 5, 9, 11, 16, 17, 21 and 24) were selected which is shown in Fig. 1. In this study, for the selection of theses 9 wards among 31, Natural Breaks (Jenks) method through ArchGIS has been implemented.

### 2.2 Sampling process

**Fig. 2:**
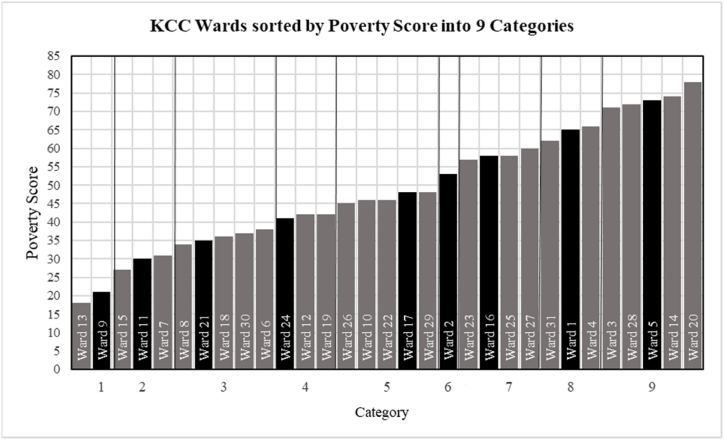
KCC wards sorted by Poverty Score into 9 categories (selected wards for sampling are indicated in black).

To understand waste generation rates and waste composition throughout the study area, household waste is collected in representative wards and in households. Although the correlation between waste generation and income level is well described [26], other socioeconomic or cultural factors might also play a role. In order to implicitly incorporate a large number of different parameters into the selection process, this study uses the UNDP Poverty Score to determine a representative selection of wards for the waste sampling process. The UNDP Poverty Score indicates significant differences in terms of multidimensional poverty between the wards ranging from a score of 18, in Ward 13 to a score of 78 in Ward 20 (see also Fig. 2). In total, the UNDP Poverty Score incorporates 16 indicators covering infrastructure access, access to education, employment status, social problems, or income level [27]. Although the Poverty Score is correlated to income levels, it provides a broader context of the living situation in the different wards. By applying the Natural Break method, on the Poverty Score, the wards are classified into a defined number of categories. The Natural Break method is a standard classification method for geospatial datasets which maximizes differences between groups while minimizing differences within groupings and allows to identify natural groupings within datasets. For this study, the number of categories was set to nine, as a compromise between sampling effort and maximal coverage. From each category one ward was randomly selected. The selected wards are 1, 2, 5, 9, 11, 16, 17, 21 and 24. Fig. 2 shows the Poverty Index in the 31 wards of KCC and the selected wards.

To determine the sample size, practitioner guidelines for household waste analysis [28] recommend to obtain a 1%-sample of the weekly generated waste. With an estimated average weekly waste generation of 105 tons per ward, the goal is to retrieve a 1050 kg-sample for each selected ward. Assuming an average household size of five, this amount can be generated by 75 households. Consequently, the study aims for an overall sample size of 9450 kg of waste from 675 households across nine wards.

Household income is a determining factor in waste generation and waste composition [26]. Therefore, the actual sampling is based on income level. For this, a preliminary survey was carried out in order to determine the socioeconomic situation. To gather information on socioeconomic and daily solid waste characteristics at the household level, a structured questionnaire was developed, pre-tested, and modified. Five different socioeconomic groups based on monthly household income were identified: low-income, lower-middle-income, middle-income, higher middle-income and high-income (see Table 1). With a total sample size of 75 households per ward, each income group is represented by 15 households which are randomly chosen.

**Table 1:** Categorization of income group for selected wards.

Socio-economic condition	Income (BDT)	Income (USD) 100BDT = 0.92USD	No of households sampled
Low-income families	<10,000	<92	15
Lower-middle-income families	10,000–20000	92–184	15
Middle income-families	20,000–30000	184–276	15
Higher-middle-income families	30,000–40000	276–368	15
Higher-income-families	>40,000	>368	15
Total			75

The study area's residents were visited individually to notify them of the survey and to determine their willingness to participate. A structured questionnaire was used to conduct a formal survey of household residents, collecting data on the current waste management practices and socioeconomic conditions of the residents. Trained investigators and data collectors conducted house-to-house interviews using the questionnaire to gather data on waste management knowledge. After the questionnaire survey, two types of bins were provided to the selected households and trained on how to store, sort, and separate waste into different bins. Two different labeled bins were provided for departing different types of waste. One for biodegradable waste and another for non-biodegradable waste.

### 2.3 Waste collection and laboratory test

**Fig. 3:**
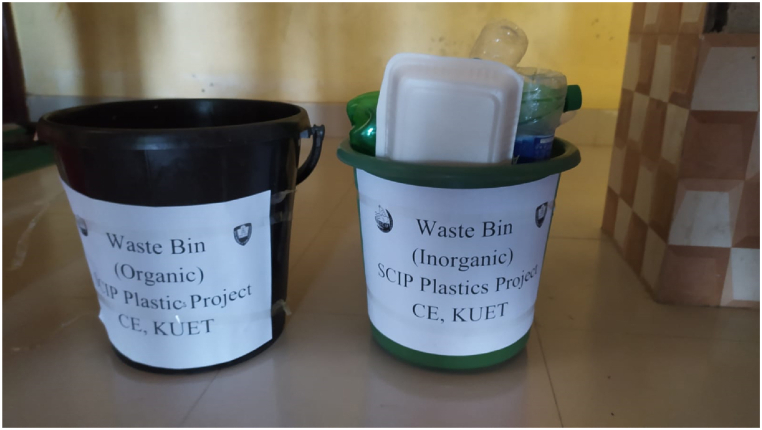
Household solid waste storage bin for organic and inorganic waste (Source: Author, 2022).

The selected households are provided with two types of waste bins: one biodegradable and the other non-biodegradable as shown in Fig. 3. Household waste from each household is collected every other day over a 14-day period to obtain a sample for every day of the week. A sampling day is followed by a day for analysis. The collected waste from each household is weighed, recorded and transported to the laboratory. In the laboratory, the physical parameters waste composition, moisture content and density of the municipal waste are determined. In this study, the density of municipal waste was determined by filling a specified volume and mass container with a waste sample and then weighing the filled container. In addition, the container of waste was constantly shaken during the filling of municipal waste. Then, the density is calculated by dividing the net weight of the solid waste sample by the container's volume is shown in eq. (1). For determining the bulk density of municipal waste, the working procedure of Alabdraba and AL-Qaraghully [29] was followed

(1) $$D=\frac{M}{V}$$

Here, D = density of solid waste (Kg/m3).

M = Net weight of waste samples (kg)
V=Volume of container (m3)

The moisture content was determined by weighing the wet sample which represented the waste sample and then drying it to a constant weight at a temperature of 105 °C and then measuring the weight change. But, in the case of plastics, the temperature was fixed at 60 °C. This weight loss is then expressed as a percentage that represented moisture content shown in eq. (2).

(2) $$M=\frac{A-B}{A}$$

(eq. 3) M=(A-B)/A ×100 … … …

M = Moisture content of solid waste (%)
A = Initial weight of waste samples (kg)
B=Weight of waste samples after dried (kg)

### 2.4 Waste sorting and analysis

**Fig. 4:**
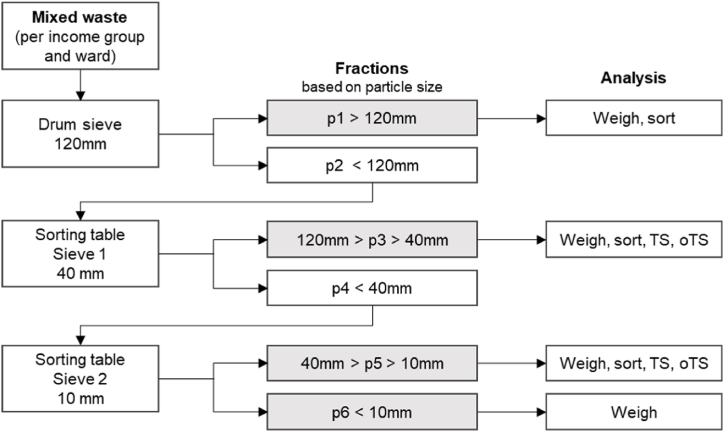
Waste sorting process resulting in four size fractions for further analysis (p: particle size, TS: total solids, oTS: organic total solids).

**Fig. 5:**
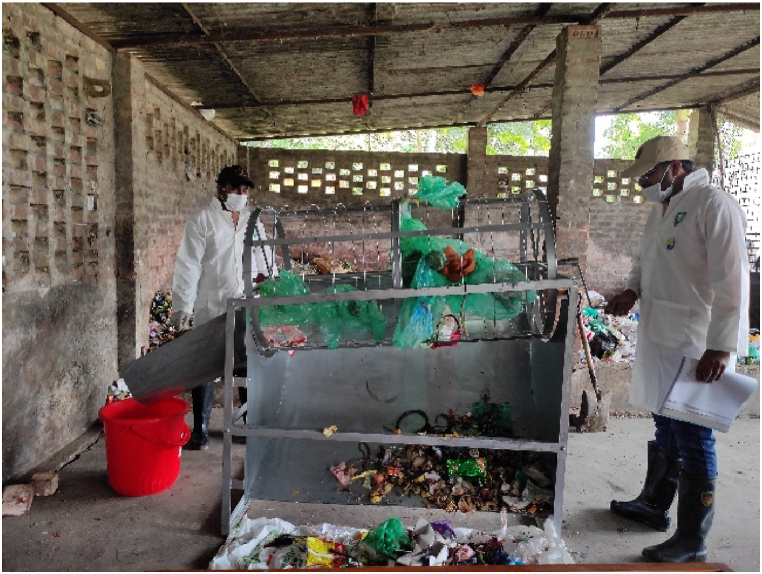
Sieving waste sample through rotary drum sieve (PC: Author, 2022).

**Fig. 6:**
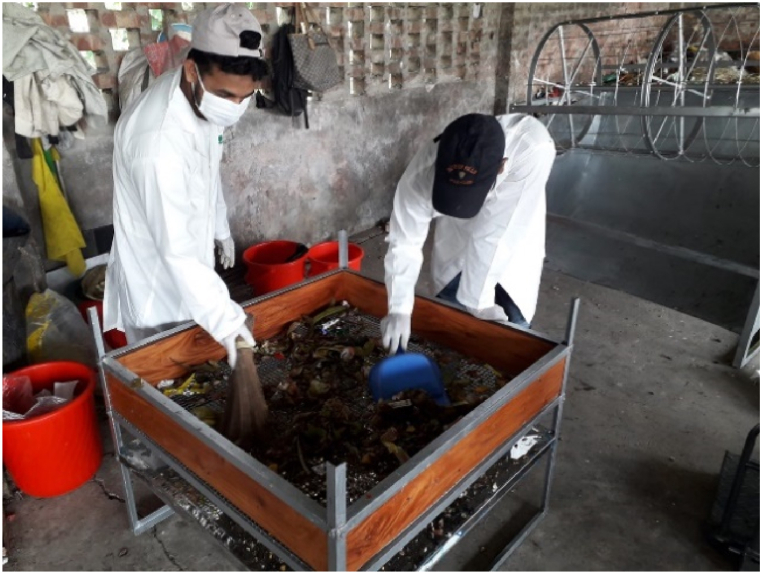
Sieving waste sample through individual modular screens in sorting table (PC: Author, 2022).

The collected waste samples are based on income group and ward, resulting in ten sub-samples (five organic and five inorganic) per ward according to the defined income groups. Each sub-sample goes through three screening stages: a 120 mm drum sieve and two flat screens on a sorting table, the first one with a 40 mm screen and the second with a 10 mm screen (see Fig. 4, Fig. 5, Fig. 6). This process results in four size fractions: particles larger 120 mm (p1), particles smaller 120 mm and larger 40 mm (p3), particles smaller 40 mm and larger 10 mm (p5) and the fine fraction with particles smaller 10 mm (p6) which is classified as ash and dust.

The size fraction p1, p3 and p5 are then sorted by material into eight categories: (1) organic waste, (2) plastics, (3) paper & cardboard, (4) glass, (5) textiles & wood, (6) electrical goods incl. batteries, (7) metals, (8) medical/sanitary waste. Each fraction is weighed. The final dataset results initially in information by income group on waste generation rates in weight per capita and day and waste material composition in weight percentages. Furthermore, the data contains information on particle size distribution, moisture content and bulk density of selected fractions.

### 2.5 Data extrapolation and total waste generation

The datasets from the 75 households are extrapolated to ward level based on income distribution. Since there are no official records on income distribution within KCC, this study comprises a survey to determine the income distribution in the nine ward categories. The required sample size is calculated by means of total population size in the wards, a defined margin of error and confidence level using the following equation (Eq. 3.)

(4) $$n=\frac{\frac{Z^{2}p\left(1-p\right)}{e^{2}}}{1+\frac{Z^{2}p\left(1-p\right)}{e^{2}N}}$$

Where: n = Sample size (being determined); N = Population size; p = the proportion in the target population estimated to have characteristics being measured (0.5); e = 0.05 (since the acceptable error should be 5 %) and z = Standard deviation at a given confidence level (z = 1.645 at 90 % CL).

Table 2 shows the total population and number of households of the ward categories, as well as the calculated required sample. To simplify the survey, the number of surveyed households was set to 270. Households are randomly chosen throughout each ward and housing typology is used as a guideline to identify different income levels in the field.

**Table 2:** Required and actual sample size to determine income distribution in selected ward (ward categories) based on demographic information in wards.

Category	Ward No	Total Population	Total Household	Sample Size	Surveyed HH
1	*9*	45,700	9920	266	270
2	*11*	27,500	5970	261	
3	*21*	36,760	7790	264	
4	*24*	57,500	12,895	267	
5	*17*	65,320	14,580	268	
6	*2*	36,000	8145	264	
7	*16*	61,000	12,890	267	
8	*1*	37,250	8280	264	
9	*5*	25,350	5340	261	

The resulting datasets provides the percentage of people in each income group and consequently, the absolute number of people in each income group can be derived. With this income distribution, the waste sampling results are projected to ward level. To calculate the category-based waste generation rate in kg per day, per-capita rates in each income group are multiplied with the calculated absolute number of people in the income group and summed up. The income distribution is assumed to be the same within a ward-category.

### 2.6 Data analysis

Correlation analysis is applied to understand the relationship between two continuous variables in a dataset. It is commonly used in various fields and situations where there is a need to explore the association between two variable. In this study, the correlation analysis is used to obtain the correlation coefficients of waste generation with family income, family size, education level and average age of family members. The strength of the correlation between the variables was found using the IBM SPSS Statistic (Statistical Package for Social Sciences) as a processing software application. To screen out potentially significant variables, univariate binary logistic regression analysis was done. Utilizing the identified variables, a multivariable binary logistic regression analysis was conducted to examine the connection between the dependent variable and independent variables at a significance level of 5 %. Multivariable binary logistic regression analysis was done at 95 % confidence interval and variables with a P-value<0.05 were considered as statistically significant.

According to Supangkat and Herdiansyah [30], the criteria between two variables need to meet the following criteria.

0.00–0.199: Very weak correlation.

0.20–0.399: Weak correlation.

0.40–0.599: Medium correlation.

0.60–0.799: Strong correlation.

0.80–1.0: Very strong correlation.

The correlation between two variables was considered statistically significant if the p-value is lower than 0.05 and the correlation between two variables was considered not significant if the p-value is greater than 0.05.

## 3 Results and discussion

### 3.1 Waste generation analysis

Based on the income survey in the selected wards the income distributions were determined for the nine ward categories. They are presented in Table 3. The highest percentage of low income and lowest percentage of high-income households are found in Category 8 (Ward 1) with 23.7 % low-income households and 10.37 % high income households. Category 5 (Ward 17) shows the lowest percentage of low-income households with 9.63 %, and the highest percentage of high-income households with 24.81 %.

**Table 3:** Income distribution in selected ward categories.

Income distribution (%)									
Category	1	2	3	4	5	6	7	8	9
Ward	9	11	21	24	17	2	16	1	5
Low	14.81	16.3	14.81	13.7	9.63	20	18.15	23.7	21.85
Lower Middle	23.7	24.44	23.7	20.74	21.85	26.3	23.33	27.78	27.41
Middle	22.59	25.56	23.33	20	22.59	24.44	23.7	25.93	26.67
Higher Middle	21.85	19.26	24.44	24.81	21.11	16.3	18.15	12.22	13.33
High	17.04	14.44	13.7	20.74	24.81	12.96	16.67	10.37	10.74
Total	100	100	100	100	100	100	100	100	100
Total Pop	45,700	27,500	36,760	57,500	65,320	36,000	61,000	37,250	49,850

As expected, there is a strong correlation between waste generation rates and income level (r = 0.997). Table 4 provides the waste generation rates (WGR) in kg per capita and day in the different ward categories. The WGR ranges from 0.76 kg/(cap‧d) in Category 5 (Ward 17) in the high-income group to only 0.27 kg/(cap‧d) in Category 8 (Ward 1) in the low-income group.

**Table 4:** Waste generation rate in the different ward categories in kg per capita and day.

	**Waste generation rate** [kg/(cap‧d)]										
**Category**	**1**	**2**	**3**	**4**	**5**	**6**	**7**	**8**	**9**	**Average**	**SD**
Ward	9	11	21	24	17	2	16	1	5		
**Low**	0.340	0.330	0.310	0.320	0.330	0.300	*0.320*	0.270	0.290	0.312	0.021
**Lower Middle**	0.390	0.400	0.390	0.380	0.440	0.350	*0.380*	0.330	0.360	0.380	0.030
**Middle**	0.550	0.530	0.490	0.450	0.520	0.430	*0.450*	0.390	0.410	0.469	0.053
**Higher Middle**	0.670	0.630	0.550	0.510	0.680	0.520	*0.570*	0.490	0.500	0.569	0.070
**High**	0.740	0.710	0.690	0.600	0.760	0.640	*0.620*	0.560	0.550	0.652	0.072
**Average**^a^	0.540	0.511	0.482	0.464	0.536	0.425	*0.490*	0.375	0.397	0.469	0.056
**SD**	0.148	0.130	0.116	0.093	0.148	0.110	*0.106*	0.092	0.140	0.120	

a Geometric mean based on income distribution.

**Fig. 7:**
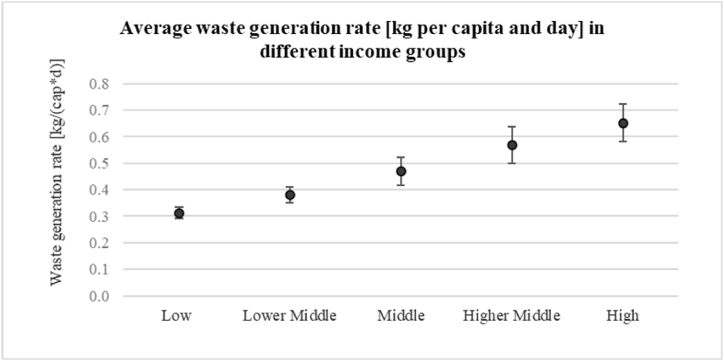
Average waste generation rates and standard deviation in different income groups.

On average, low-income households generate daily 0.312 kg/cap, middle-income households 0.469 kg/cap and high-income households 0.652 kg/cap. Among the nine categories the standard deviation within each income group is low (≤0.07 kg/(cap‧d)) indicating low dispersion within each group. However, the standard deviation increases with the income level (see also Fig. 7), which might indicate that in high-income-households additional factors play a role in waste generation.

**Fig. 8:**
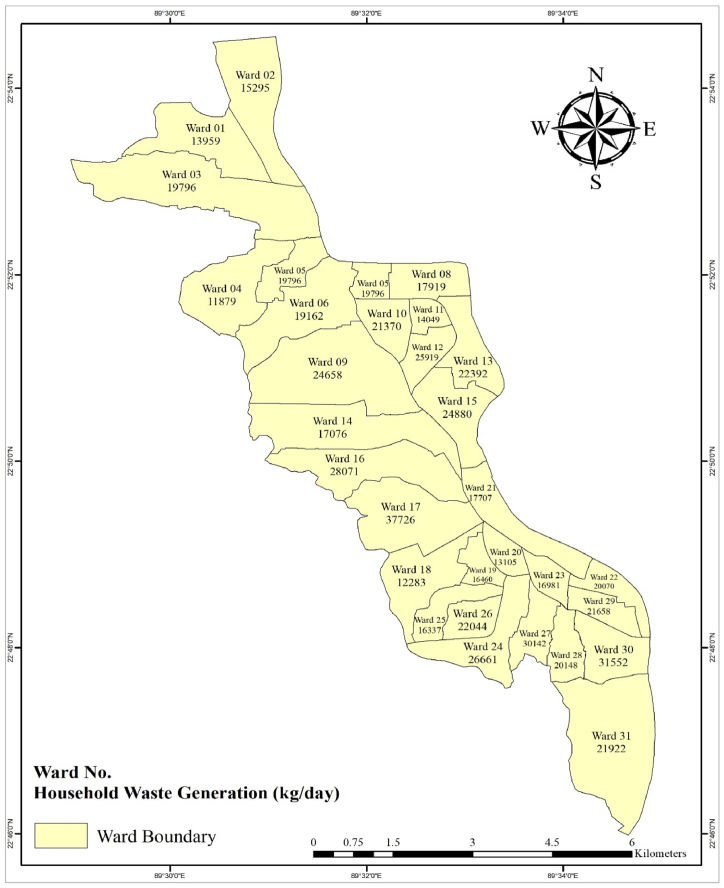
Absolute waste generation in all 31 KCC wards (projected data) in kg per day.

By means of the income distribution the waste generation data is extrapolated to each ward category and finally to the whole Khulna city area. Fig. 8 illustrates the varying estimated waste generation rates throughout the KCC wards. According to this study's findings, Wards 4, 18 and 20 produce the lowest amounts of waste, whereas Wards 17, 16 and 27 produce the largest amount of waste with up to 35 metric tons per day. In total, KCC households produce approximately 641 metric tons per day. This information is critical for the implementation of waste collection services and design of waste management facilities throughout the city. It allows for efficient resource allocation within the city. By knowing the amount of waste generated in each ward, city authorities can allocate resources such as waste collection vehicles, manpower, and equipment based on the specific needs of each ward. This ensures that waste management services are adequately provided to all areas, optimizing efficiency and reducing operational costs.

However, it was estimated that, the waste generation in 6 (six) major urban areas of Bangladesh viz. Dhaka, Khulna, Chittagong, Rajshahi, Barisal and Sylhet was 0.70, 0.47, 0.56, 0.27, 0.44 and 0.25kg/cap/day, respectively [31]. The present study is close to the researcher Mostakim et al. (2021).

### 3.2 Effect of the weekend on waste generation rate

The waste generation rate is also influenced by the weekends of all income-level households. The MSW generation rate of different socioeconomic levels over 7 days (both weekdays and weekends) for Ward 24 is presented in Table 5. In Bangladesh, all the government and non-government offices and institutions remain closed on Friday. Besides, all government and a few non-government offices and institutions pass Saturday as a holiday. It was observed that the waste generated rate differs during the weekdays, with the highest quantities generated and collected on Saturday. This trend was noticed in every socioeconomic group. The cause for this was revealed to be due to Friday's activities, which is considered a holiday that most people spend with their families. As they stay at home, waste generation is greatly affected. On the other hand, the waste collector collects household waste in the morning. The waste volume and weight were greater on Saturday in every income group. The result shows, the waste generation rate on Saturday was 0.51 kg/cap/day. In addition, Saturday is also a holiday for some government and non-government offices and institutions. So, Saturday's waste which was collected on Sunday was comparatively higher than other weekdays and the waste and it was found 0.46kg/cap/day. The researcher Letshwenyo and Kgetseyamore [32] conducted a study on waste generation and its characteristic at Palapye city, Botswana. They estimated that, the weekend plays an important role to increase the waste generation in a municipality. This study also express the same finding of Letshwenyo and Kgetseyamore [32].

**Table 5:** Waste generation rate of different socioeconomic levels over 7 days (both weekdays and weekends).

Status	Waste generation rate (kg/cap/day)						
	Thursday	Friday	Saturday	Sunday	Monday	Tuesday	Wednesday
Low	0.30	0.31	0.37	0.34	0.32	0.33	0.29
lower middle	0.36	0.38	0.42	0.39	0.37	0.36	0.37
Middle	0.44	0.44	0.51	0.44	0.42	0.44	0.43
Higher middle	0.50	0.52	0.56	0.53	0.49	0.49	0.49
High	0.58	0.58	0.67	0.60	0.61	0.58	0.58
Average	0.44	0.45	0.51	0.46	0.44	0.44	0.43

### 3.3 Characteristics of solid waste

#### 3.3.1 Waste composition in each income group

**Fig. 9:**
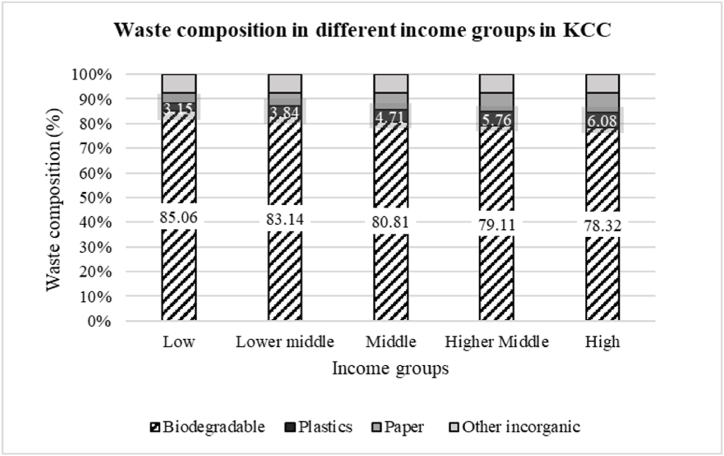
Waste composition in different income groups in KCC.

The organic, biodegradable or putrescible fraction of the waste which contain mostly plant matter or food scraps is decreasing with rising income level. Dikole and Letshwenyo [33]stated that high family income could have led to the high percentages of non-biodegradable or organic waste in the mixed waste that was collected from households. This can also be observed in the present study. Whereas low-income households produce waste with around 85 % biodegradable content, this fraction is reduced to around 78 % in high-income households. At the same time the plastic and paper fraction increases (see Fig. 9. The plastic waste fraction almost doubles from 3.15 % in low-income households to 6.08 % in high-income households.

Generally, households with higher income produce a larger proportion of paper & cardboard (7.92 %), electric goods (0.46 %) and metals (0.60 %), whereas these fractions are significant lower in waste of low-income households: 4.38 % paper & cardboard, 0.11 % electric goods, 0.12 % metals. Households with higher incomes may purchase more packaged goods or consume more processed food, which could result in higher amounts of plastic packaging and food waste. On the other hand, households with lower incomes may produce comparatively more organic waste, such as food scraps, due to their dietary habits or lack of access to processed or packaged food. As a result, With the increase of household income, the proportion of biodegradable and textile waste gets down due to inhabitant habits. The key finding of the study is that as household income increases, there is a decrease in the proportion of organic, biodegradable, or putrescible waste in the overall waste generated. This decrease in organic waste is accompanied by an increase in the proportion of non-biodegradable waste, such as plastic and paper, particularly in high-income households. The study suggests that higher family income may lead to a shift in waste composition, with households producing more plastic and paper waste and relatively less organic waste. This change in waste composition is attributed to differences in dietary habits, access to processed or packaged food, and purchasing behavior between households with different income levels.

#### 3.3.2 Composition of household waste

The composition of municipal solid waste collected during the investigation that is generated in Khulna City at different wards is presented in Table 6. Knowing the composition of the generated waste is a significant factor that can aid in the development of effective strategies for reusing, recycling, storing, processing, transporting, and ultimately disposing of the waste. The collected solid wastes were composed of biodegradable waste, plastics, glass, paper & cardboard, textiles &wood, electric goods, metals, ceramic, medical waste, dust and others. The characteristics of household waste generated from the selected 75 households over 7 days show that the largest category of waste in the overall waste stream is biodegradable waste, which makes up 81 % of the total waste. The wards 1, 2 and 5 generate maximum biodegradable waste more that 82 % of total waste. Plastics, paper and cardboard, and dust and others are the next largest categories of waste. In the study area, ward 16, 17, 21 and 24 generate nearly 5 % plastic. The remaining categories of waste, including glass, textiles and wood, electric goods, ceramic, metals, and medical waste, make up relatively small proportions of the overall waste stream, ranging from 0.21 % to 1.55 %. This waste composition value was extrapolated and determined the total quantity of each waste fraction that are generated at KCC.

**Table 6:** Average waste composition of the selected wards (wet weight).

	Weight Percentages (%)									SD
Composition	Ward 1	Ward 2	Ward 5	Ward 9	Ward 11	Ward 16	Ward 17	Ward 21	Ward 24	
Biodegradable Waste	82.29	82.06	82.76	81.89	81.84	80.81	81.29	80.85	80.76	0.719
Plastics	4.33	4.44	4.34	4.21	4.22	4.71	4.71	4.76	4.77	0.222
Paper & cardboard	5.96	6.16	5.55	6.22	6.23	6.90	6.47	6.91	6.92	0.472
Glass	0.65	0.65	0.73	0.66	0.66	0.65	0.67	0.64	0.63	0.024
Textiles &wood	1.68	1.64	1.53	1.47	1.65	1.35	1.59	1.57	1.5	0.104
Electric goods	0.29	0.30	0.32	0.31	0.30	0.35	0.31	0.35	0.35	0.023
Ceramic	0.69	0.70	0.72	0.90	0.72	0.84	0.64	0.62	0.69	0.090
Metals	0.33	0.35	0.37	0.36	0.37	0.44	0.37	0.44	0.44	0.043
Medical waste	0.20	0.21	0.22	0.20	0.21	0.23	0.21	0.23	0.23	0.010
Dust & others	3.58	3.49	3.48	3.78	3.80	3.72	3.75	3.63	3.71	0.122

#### 3.3.3 Projected results: waste composition in the different wards

**Fig. 10:**
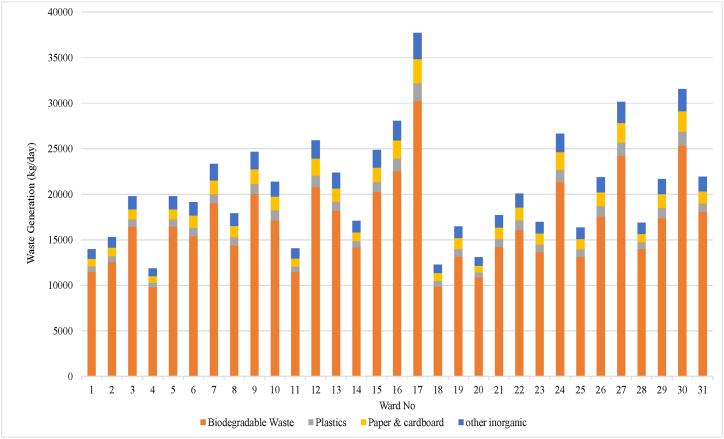
Waste generate in different wards in KCC.

**Fig. 11:**
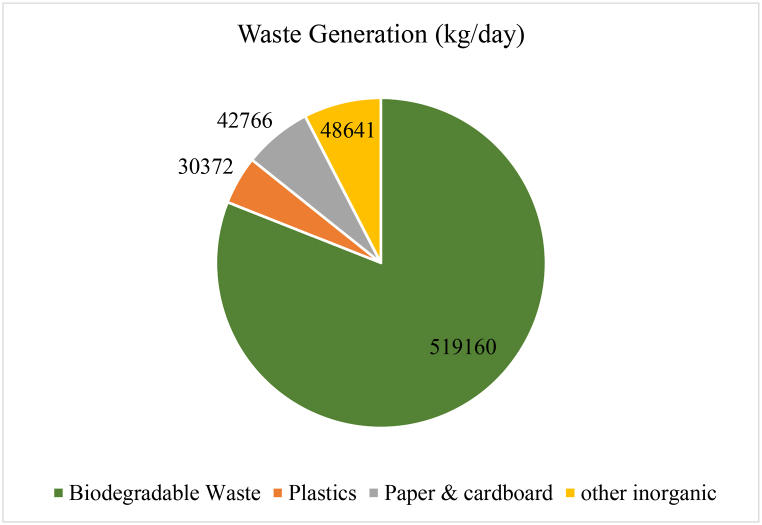
Waste composition in different income groups in KCC.

By means of the waste generation the waste composition data is extrapolated to each ward category and finally to the whole Khulna city area. Fig. 10, Fig. 11 show the total waste and its fraction that are generated at 31 wards in KCC. Maximum waste is generated at ward 17 due to high rate of waste generation waste and high population. It is estimated about 30,255 kg biodegradable waste, 1957 kg plastic waste, 2654 kg paper & cardboard and 2859 kg other inorganic waste are produced in ward 17. High-income families have more purchasing power and are often able to afford a wide range of products and services. Consumerism drives the production and consumption of goods, leading to more waste generation [34]. In ward no 17, the higher percentages of high income families are seen. The population of that ward is also high. So, the waste like biodegradable, plastic and paper production in that ward is comparatively higher than in other wards. The result shows that the lowest waste was generated at wards 4, 18, and 20.

The result shows that approximately 81 % waste are biodegradable waste which amount is about 519160 kg/day (Fig. 11). The high organic content in the waste means that leachate is produced whenever the waste is stored for a while. It means odor and pest problems. It also means that a certain amount of weight is lost at the transfer stations because water will evaporate or leak away. It also means that transporting the organic fraction might have the main impact on transport costs in the waste management system, because organic waste is heavy. Whereas paper and plastic waste could be compacted and transported over longer distances this is not feasible for organic waste. Here, the transport route should be as short as possible. The amount of plastic and paper is also significant. It was estimated that 30,372 kg plastic, 42766 kg paper are produce per day. This huge amount of plastic and paper could be turn into recycle and minimize plastic pollution.

**Fig. 12:**
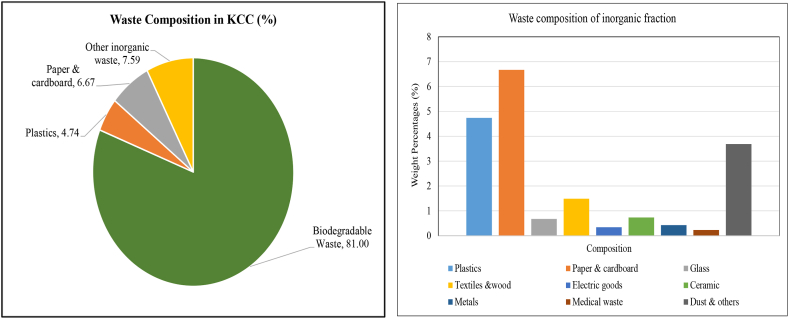
Waste composition in KCC.

Fig. 12 shows the average waste composition in Khulna City Corporation. After extrapolating the household data, the overall waste composition was measured. The finding reveal that, the biodegradable waste was the prominent type of waste and its percentage was 81 %. Plastics and paper & cardboard are the next largest categories of waste, making up 4.74 % and 6.67 % of the total waste, respectively. The remaining categories of waste, including glass, textiles and wood, electric goods, ceramic, metals, and medical waste, make up relatively small proportions of the overall waste stream, ranging from 0.21 % to 1.55 %.

#### 3.3.4 Comparison of between present study and literature

**Fig. 13:**
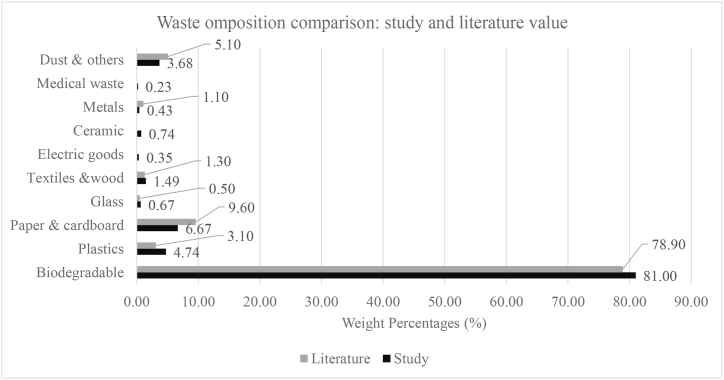
Comparison of physical composition between the present study and literature.

The Comparison of major components between the present study and the literature is shown in Fig. 13. Researchers Ahsan [3]conducted a study to characterize the municipal waste of six major cities in Bangladesh. They found 78.9 % organic matter, 9.5 % paper & cardboard, 1.3 % textiles and 0.5 % glass. The percentage of plastics in the mixed waste was also remarkable and its proportion was 3.1 %. Plastics are comparatively very chip and easy to carry from one place to another. In addition, they have useful properties such as being waterproof or semi-permeable. So, the inhabitants feel more comfortable using plastic rather than other products. Therefore, the present study showed comparatively high percentages of plastics and its value is about 4.74 % while paper & cardboard is 6.67 %.

#### 3.3.5 Particle size distribution

**Fig. 14:**
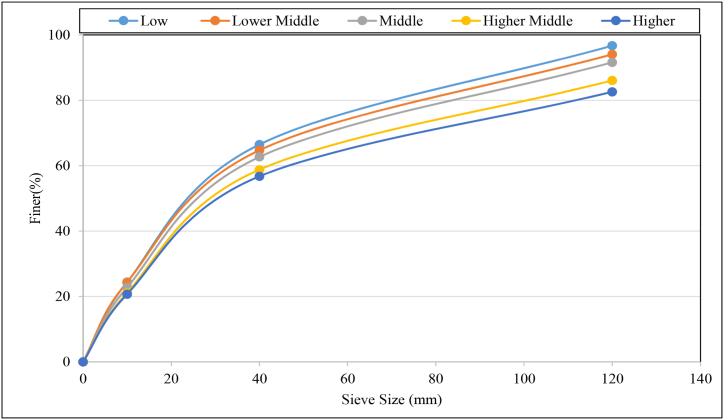
Particle size distribution of municipal solid waste for different economic groups.

Fig. 14 shows the size distribution of municipal solid wastes that are generated in ward No 24 of selected different socioeconomic households. At first, the waste samples were sieved through a rotary drum sieve that has a square opening of 120 mm. After that, the sieved wastes through 120 mm were passed into the individual modular sieve that has two square sieves of 40 and 10 mm. The trend of particle size distribution is found as a gradual decrease for the smaller sieve openings. Family income also affects the particle size of waste. Result reveals the percentages of finer decrease with the increase of family income. For high-income families, finer was obtained 82.58 % which increased to 96.72 % for low-income family (Fig. 14).

#### 3.3.6 Bulk density and moisture content in different waste fraction

When managing waste, water content and bulk density are critical factors for designing an appropriate waste collection system. Low bulk density leads to large volumes where garbage compactors might play an important role in an efficient waste collection service. However, high bulk densities might rather lead to the failure of compactors. On the other hand high water content which goes generally hand in hand with high bulk density needs to be carefully managed throughout the collection process and especially at the final disposal site, it the water content will directly contribute to leachate production and thus potentially pose an environmental threat. 7 shows the moisture content and bulk density of waste components of the selected area. Results reveal that biodegradable waste has the highest water content with 74.86 % and at the same the same time the highest bulk density with 553.8 kg/m^3^. Plastic waste on the other hand shows the lowest water content and bulk density with 16.54 % moisture content and 43.62 kg/m^3^ density. Researchers Alamgir and Ahsan [35]stated that, the moisture content of municipal solid waste ranges from 56 to 75 % for Bangladesh. Besides, Bulk densities were determined in 3 states of compactness as described earlier. In loose state, bulk density ranges from 549 to 669 kg/m^3^, while in the medium state, it ranges from 764 to 951 kg/m^3^ and for compacted state 875–1127 kg/m^3^. The finding of this study is also in range of the finding of the Researchers Alamgir and Ahsan [35].

### 3.4 Relationship among waste generation, socioeconomic factors income level and waste composition

#### 3.4.1 Relationship between waste generation and socioeconomic factors

The correlation between waste generation and different socioeconomic parameters such as household income level, family size and education level are presented in Table 8. The correlation study disclosed that the waste generation of a community is positively correlated with the family income ((r = 0.997, p < 0.05) which indicates the waste generation of a household is increasing with increasing income level. Sankoh [36]also stated that a positive relationship existed between waste generation at households and income levels.

**Table 7:** Results of moisture content and bulk density of waste components.

Fraction	Moisture content (%)	Bulk density (kg/m^3^)
Biodegradable Waste	74.86	553.8
Plastics	16.54	43.62
Paper	27.12	112.38
Glass	–	295.91
Textiles & woods	32.17	126.32
Electric Goods	–	133.72
Metals	–	438.41
Dust & others	33.61	411.17

**Table 8:** Relationship between socioeconomic factor and waste generation.

Socioeconomic Factor	Correlation with waste generation	
	Correlation coefficient (r)	P value
Income	0.997	0.00018
Family Size	0.845	0.2022
Education Level	O.527	0.0230
Average age (years) of family members	−0.227	0.416

Many research findings indicated that the generation of waste tends to rise as income increases. The number of household members is the most important factor influencing this correlation. For example, some families had a high income but produced little waste. This situation could be caused by the fact that the family has fewer members. In this study, a positive correlation (r = 0.845, p > 0.05) was observed between waste generation and family size though the relation is not statistically significant. There could be several explanations for the non-significant correlation. It could have happened as a result of taking food from outside and reusing some types of waste. The education level also plays an important role to generate waste at the household level. Findings reveal that the generation of waste in the household in positively correlated with education level though the relation is not statistically significant. Trang et al. [37] conveyed that waste generation is positively correlated with education level. The findings of this study is well agreed with the postulation of Trang et al. [37]. In addition, the average age of a family can have an impact on waste generation in several ways. Generally, younger families with children tend to produce more waste compared to older families or households with no children. This is because younger families often have more disposable income to buy products and tend to consume more packaged and convenience foods. On the other hand, older families or households with no children may generate less waste because they may have more established routines and may be more conscious of waste reduction efforts. Therefore average age of family members is negatively correlated (−0.227) with waste generation.

#### 3.4.2 Correlation between income level and waste composition

Table 9 illustrates the correlation between income level and different types of waste that are generated in the study area. The result reveals that there were positive correlations between monthly income and the types of waste generated such as Plastics (r = −0.990, p < 0.05), Paper & cardboard (r = 0.975, p < 0.05), Glass (r = 0.248, p > 0.05), electric goods (r = 0.911, p > 0.05), Metal (r = 0.979, p < 0.05), Medical waste (r = 0.979, p < 0.05). Positive correlation donated that with the increasing in family income increase the plastic, paper & cardboard, glass, electric goods, metal and medical waste generation. But, glass and electric goods, were not significant at 5 % level. An increase in income leads to an increase in the consumption of goods, which may have resulted in an increase in plastic, glass and metal waste generation.

**Table 9:** Correlation between income level and waste composition.

Waste composition	Correlation with income level	
	Correlation coefficient, r	P value
Biodegradable Waste	−0.987	0.0016
Plastics	0.990	0.0011
Paper & cardboard	0.975	0.0046
Glass	0.848	0.0691
Textiles &wood	−0.756	0.1390
Electric goods	0.911	0.0312
Metals	0.979	0.0037
Medical waste	0.973	0.0053
Dust & others	−0.870	0.0554

Sivakumar and Sugirtharan [38] stated that family income is positively correlated with the generation of plastic, paper, glass and metal. A negative correlation also observed the biodegradable waste (r = 0.987, p < 0.05), Textiles and wood (r = 0.756, p > 0.05) and dust (r = 0.870, p > 0.05) (Table 9). It indicates that as family income level increases the generation of biodegradable waste, textiles and dust decreases.

### 3.5 Source separation potential of recyclable waste

For evaluating the potential of source separation, 20 households (4 households from each income group) were selected. Two types of waste (e.g. source separated waste and mixed waste) were collected from the selected household. After collecting waste from the selected households, the waste collector of the selected area was advised to sort out the wastes which are generally separated by themselves during waste collection from households. The source separation potential of recyclable waste is described in the following section.

#### 3.5.1 Waste composition of source-separated MSW before and after sorting

After collecting waste from the selected households, the waste collector of the selected area was advised to sort out the wastes which are generally separated by themselves during waste collection from households. The waste collector willingly separated the materials that have value in the markets. Then the physical composition of the collected waste was determined to know the source separation potential of recyclable waste. The waste composition of source-separated collected waste before and after sorting by a waste collector is presented in Table 10. The result reveals that waste composition differs between before and after waste composition. The plastic fraction in the source-separated wastes was found 4.69 and 2.99 % before and after sorting, respectively. The same reduction was observed in paper and cardboard, metals and medical waste. Interestingly, all metals were separated and collected by the waste collector. Paper and cardboard were found 6.55 and 3.92 % before and after sorting, respectively (Table 10).

**Table 10:** Waste composition of source-separated MSW before and after sorting by waste collector.

Waste composition	Source separated waste		Mixed waste	
	Before sorting by waste collector (%)	After sorting by waste collector (%)	Before sorting by waste collector (%)	After sorting by waste collector (%)
Biodegradable Waste	81.18	85.57	81.25	83.11
Plastics	4.69	2.99	4.74	4.04
Paper & cardboard	6.55	3.92	6.61	5.48
Glass	0.64	0.67	0.69	0.71
Textiles &wood	1.27	1.34	1.20	1.22
Electric goods	0.30	0.10	0.28	0.24
Ceramic	1.04	1.10	1.14	1.16
Metals	0.32	0.00	0.33	0.24
Medical waste	0.20	0.13	0.21	0.18
Dust & others	3.81	4.18	3.55	3.63
Total	100	100	100	100

#### 3.5.2 Waste composition of mixed MSW before and after sorting

The mixed waste was also collected from the same households which were selected for source-separated waste. The waste workers were advised to sort out all the materials that are generally separated by them when they collect the mixed waste from a household. Table 10 shows the waste composition of mixed MSW before and after sorting by the waste collector. The result discloses that the valuable waste materials such as plastics, paper and cardboard, electric goods, metals and medical waste were separated by the workers. The plastic paper and metals fractions were found 4.74, 6.61 and 0.33 %, respectively before sorting the waste. These percentages were reduced after sorting and found 4.04, 5.48 and 0.24 % for plastics, paper and metals, respectively (Table 10).

#### 3.5.3 Comparison between source-separated waste and mixed waste

**Fig. 15:**
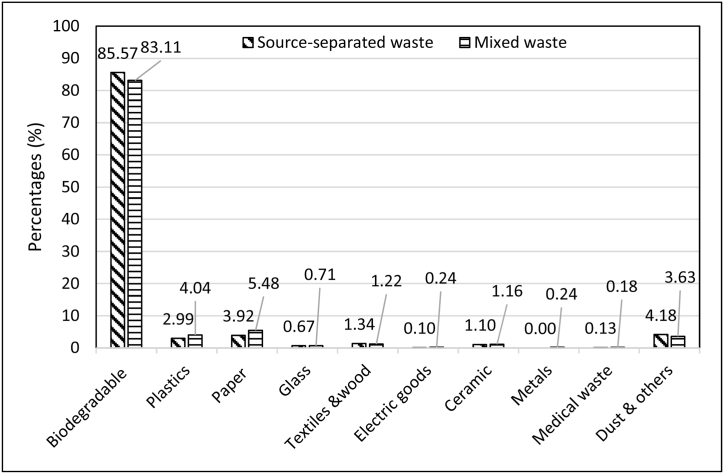
Comparison of waste composition between source-separated waste and mixed waste after sorting by the waste collector.

Fig. 15 reveals the comparison of waste composition between source-separated waste and mixed waste after sorting by the waste collector. In Khulna city, the waste collector collects mixed waste from households. But, the mixed waste collection system reduced the potential to recover recyclable materials. In this study, the comparison between mixed waste and source-separated waste for recovering recyclable materials was determined. Results expose that the proportions of plastic, paper and metals in the mixed waste were calculated 4.04, 5.48 and 0.24 %, respectively. But, this proportion can be reduced by developing a source-separated waste collection system. In the mixed waste, the organic portion mix-up with valuable materials such as paper, plastic, textiles, etc. and reduce the potential to recover the recyclable materials. Therefore, the proportion of plastics, paper and metals in the source-separate waste were comparatively lower than the mixed waste and found 2.99, 3.92 and 0 %, respectively (Fig. 15).

### 3.6 Socioeconomic factors

The distribution of the household in the surveyed area by religion, family members, education level, family employment and age of the respondents is presented in Table 11. The size of a family can have a significant impact on the amount of waste generated. Generally, larger families tend to produce more waste than smaller ones. This is because more people in the household means more consumption and therefore more waste. Most of the selected families have between 4 and 6 members, which is around 47.56 % of total respondent households (675 households). Household members less than 4 in number have been counted as 36.14 %. In addition, 16.30 % households consist of more than 6 family members. Table 4 reveals that the majority of respondents, specifically 90.7 %, identified as Muslim, while the remaining 9.3 % identified as Hindu.

**Table 11:** Socioeconomic factors that affect the quantity of solid waste generated.

Types	Range	Frequency									Total	Percentages
		Ward1	Ward 2	Ward5	Ward9	Ward11	Ward 16	Ward 17	Ward 21	Ward 24		
Religion	Muslim	70	67	65	67	72	69	69	68	66	613	90.81
	Hindu	5	8	10	8	3	6	6	7	9	62	9.19
	Total	75	75	75	75	75	75	75	75	75	675	100.00
Family Members	Less than 4	29	31	24	26	28	27	28	25	26	244	36.14
	Between 4 and 6	31	33	38	37	32	44	32	37	37	321	47.56
	More than 6	15	11	13	12	15	4	15	13	12	110	16.30
	Total	75	75	75	75	75	75	75	75	75	675	100.00
Age of the respondent	>25	5	6	6	5	4	3	5	4	5	43	6.37
	26–35	33	29	27	25	34	27	32	24	25	256	37.93
	36–45	16	17	15	19	15	23	17	21	19	162	24.00
	46–55	9	8	9	8	10	11	9	8	8	80	11.85
	56–65	7	8	10	10	6	8	7	10	10	76	11.26
	65–75	2	3	5	5	3	2	2	5	5	32	4.74
	>75	3	4	3	3	3	1	3	3	3	26	3.85
	Total	75	75	75	75	75	75	75	75	75	675	100.00
Education Level of the respondent	Primary	26	27	25	20	16	13	26	20	20	193	28.59
	Secondary	23	21	22	22	21	21	23	22	22	197	29.19
	Higher Secondary	14	16	16	17	21	24	14	17	18	157	23.26
	Higher Education	12	11	12	16	17	17	12	16	15	128	18.96
	Total	75	75	75	75	75	75	75	75	75	675	100.00
Family employment (Person/HH)	One	56	52	55	46	48	44	47	50	49	447	66.22
	Two	10	13	14	18	15	21	21	18	17	147	21.78
	More than two	9	10	6	11	12	10	7	7	9	81	12.00
	Total	75	75	75	75	75	75	75	75	75	675	100.00

Education is a key indicator of an individual's lifestyle and social standing. It affects many aspects of life, including environmental concerns and behavior. In the study area, 29.19 % respondents have a secondary degree, 23.26 % respondents have a higher secondary degree and 18.96 % respondents are higher educated. The average age of a family can have an impact on waste generation in several ways. Generally, younger families with children tend to produce more waste compared to older families or households with no children. A large percentage (37.93 %) of respondents were aged 26–35 years (Table 11).

### 3.7 Household attitudes regarding the household waste management

Household involvement is essential for the success of any program aimed at reducing SW at its source. A well-informed and concerned public greatly facilitates and ensures the program's success. 75 households of different socioeconomic families from 9 wards (wards no. 1, 2, 5, 9, 11, 16, 17, 21 and 24) were selected for waste collection and questionnaires survey. The response of the selected household to a number of questions is shown in Table 12, Table 13 and Table 14.

**Table 12:** Household opinion on existing waste collection.

Variable Name	Types	Frequency										%	Cumulative
		Ward 1	Ward 2	Ward 5	Ward 9	Ward 11	Ward 16	Ward 17	Ward 21	Ward 24	Total		
Place for dumping	Open space	21	19	15	6	8	1	7	10	2	89	13.19	13.19
	KCC container	31	8	12	4	0	0	16	9	0	80	11.85	25.04
	Door to door	0	28	33	60	58	69	42	41	66	397	58.81	83.85
	Drain	13	11	8	0	2	0	5	7	5	51	7.56	91.41
	Hazardously	10	9	7	5	7	5	5	8	2	58	8.59	100.00
Willingness to take service from the authority	Take Service	31	36	45	64	58	69	58	50	66	477	70.67	70.67
	Don't Take service	44	39	30	11	17	6	17	25	9	198	29.33	100.00
Satisfaction regarding the service	Satisfied	24	26	32	49	44	62	42	34	51	364	53.93	53.93
	Not satisfied	7	10	13	15	14	7	16	16	15	113	16.74	70.67
	Don't take service	44	39	30	11	17	6	17	25	9	198	29.33	100.00
Frequency of waste collection	1 time	0	17	29	48	34	54	44	22	51	299	44.30	44.30
	1 time per two days	31	14	9	8	17	11	6	21	8	125	18.52	62.81
	irregular	0	5	7	8	7	4	8	7	7	53	7.85	70.67
	Don't collect	44	39	30	11	17	6	17	25	9	198	29.33	100.00
Waste collection time	6–10 a.m.	20	6	2	4	8	8	7	14	13	82	12.15	12.15
	10 a.m.-12 p.m.	0	14	17	29	31	36	31	19	22	199	29.48	41.63
	12-02 p.m.	0	11	22	19	17	20	11	6	17	123	18.22	59.85
	02–06 p.m.	6	0	1	7	1	2	3	3	3	26	3.85	63.70
	No fixed time	5	5	3	5	1	3	6	8	11	47	6.96	70.67
	Don't take service	44	39	30	11	17	6	17	25	9	198	29.33	100.00
Pay fees (BDT) (1US$ = 105BDT	0–49	0	8	9	7	9	8	13	7	21	82	12.15	12.15
	50–99	19	21	26	38	34	29	28	35	31	261	38.67	50.81
	100–150	12	7	10	19	15	32	17	8	14	134	19.85	70.67
	Don't take service	44	39	30	11	17	6	17	25	9	198	29.33	100.00

**Table 13:** Household opinion on the existing practice of waste separation.

Variable Name	Types	Frequency											
		Ward 1	Ward 2	Ward 5	Ward 9	Ward 11	Ward 16	Ward 17	Ward 21	Ward 24	Total	%	Cumulative
Waste storage system	Bin	51	55	54	64	62	71	64	54	57	532	78.81	78.81
	Polybags	18	16	15	9	10	4	8	13	17	110	16.30	95.11
	Open space	6	4	6	2	3	0	3	8	1	33	4.89	100.00
Waste storage in the bin	Mixed waste	72	74	71	75	71	75	70	69	73	650	96.30	96.30
	Separated waste	3	1	4	0	4	0	5	6	2	25	3.70	100.00
Separate recyclable waste	Yes	3	1	4	0	4	0	5	6	2	25	3.70	3.70
	No	25	21	27	21	18	22	21	17	20	192	28.44	32.15
	Some times	47	53	44	49	53	53	49	52	53	453	67.11	100.00
Use of separated waste	Reuse	3	1	4	0	4	0	5	6	2	25	3.70	3.70
	Sell	0	0	0	0	0	0	0	0	0	0	0.00	3.70
	Both reuse and sell	47	53	44	49	53	53	49	52	53	453	67.11	71.56
	Nothing	25	21	27	21	18	22	21	17	20	192	28.44	100.00
Collector of separated waste	Feriwala	47	53	44	49	53	53	49	52	53	453	67.11	67.85
	recycle shop	0	0	0	0	0	0	0	0	0	0	0.00	67.85
	Yourself	3	1	4	0	4	0	5	6	2	25	3.70	71.56
	Don't separate	25	21	27	21	18	22	21	17	20	192	28.44	100.00
How often sell	Monthly	0	2	1	4	2	5	3	2	0	19	2.81	2.81
	Bi-Monthly	7	9	9	12	7	11	8	9	2	74	10.96	13.78
	3 month	16	17	13	15	17	20	21	22	4	145	21.48	35.26
	3–6 month	24	25	21	23	27	17	17	19	47	220	32.59	67.85
	Don't separate and sell	28	22	31	21	22	22	26	23	22	217	32.15	100.00
Earn/save by selling separated materials per month (BDT)	0–50	24	25	21	23	27	17	17	19	47	220	32.59	32.59
	50–100	16	17	13	15	17	20	21	22	4	145	21.48	54.07
	100–150	7	9	9	12	7	11	8	9	2	74	10.96	65.04
	150–200	0	2	1	4	2	5	3	2	0	19	2.81	67.85
	Don't earn	28	22	31	21	22	22	26	23	22	217	32.15	100.00
Reason for not separating waste at source	Lack of awareness	25	21	24	16	15	15	17	22	25	180	26.67	26.67
	Lack of sensitivity	18	15	11	18	19	16	21	11	18	147	21.78	48.44
	Difficulty and time taking	16	17	21	16	15	22	12	17	17	153	22.67	71.11
	Mixed Waste collection system	13	21	15	25	22	22	20	19	13	170	25.19	96.30
	Separate waste	3	1	4	0	4	0	5	6	2	25	3.70	100.00

**Table 14:** Household opinion on expecting practice on waste management.

Variable Name	Types	Frequency											
		Ward 1	Ward 2	Ward 5	Ward 9	Ward 11	Ward 16	Ward 17	Ward 21	Ward 24	Total	%	Cumulative
Expected time for waste collection	6–10 a.m.	7	5	8	4	8	11	8	12	7	70	10.37	10.37
	10 a.m.-12pm	23	22	24	21	22	18	24	29	23	206	30.52	40.89
	12-02pm	35	33	31	37	27	33	31	28	35	290	42.96	83.85
	02–06pm	6	6	8	8	11	7	5	3	6	60	8.89	92.74
	Any time	4	9	4	5	7	6	7	3	4	49	7.26	100.00
Preferable distance from house to SDP (1 yd = 0.9144 m)	<250 yd	47	42	44	49	41	40	44	42	47	396	58.67	58.67
	250–500yd	28	31	26	23	34	32	29	28	28	259	38.37	97.04
	>500yd	0	2	5	3	0	3	2	5	0	20	2.96	100.00
Concern with the environment	No concern	24	27	27	19	23	19	32	33	24	228	33.78	33.78
	Have concern	49	41	45	54	48	51	40	38	49	415	61.48	95.26
	No response	2	7	3	2	4	5	3	4	2	32	4.74	100.00
The people's motivation can be increased in waste and plastic management	By TV/Radio/Newspaper	16	22	11	15	16	19	23	13	29	164	24.30	24.30
	By seminar	32	27	33	33	31	29	27	35	28	275	40.74	65.04
	Facebook/Instagram	27	26	31	27	28	27	25	27	18	236	34.96	100.00
Willing to pay if waste is collected from household	0–50	7	18	17	20	19	24	25	28	26	184	27.26	27.26
	50–100	33	31	29	37	35	42	32	22	41	302	44.74	72.00
	100–150	1	3	4	7	4	5	4	3	2	33	4.89	76.89
	Not interested to pay	34	23	25	11	17	4	14	22	6	156	23.11	100.00

#### 3.7.1 Existing waste collection system in the study area

Different NGOs and CBOs were found to be responsible for the collection and transportation of waste from households to secondary disposal points. KCC is responsible for secondary waste. They collect wastes from community bins or secondary disposal points and transport them to Rajbandh which is the final disposal site that is 10 km west of the city headquarters. The opinion of households on the existing waste collection system in the study area is presented in Table 12. Regarding the issue of where to dispose of waste, a significant proportion of the households surveyed (58.81 %) were discovered to use the services of a waste collector. Roughly 13.19 % of households discard their waste in open areas, while 7.56 % dispose of it in drains. Approximately 11.85 % of participants said they dump their waste in KCC containers, while 8.59 % of households dispose of their waste in a hazardous manner. Survey results reveal that 70.67 % of households take service from different NGOs and CBOs who collect waste door-to-door from the study area. Among the households who were interviewed, a large percentage (53.93 %) are satisfied while 16.74 % opined that they are not satisfied by the service which is provided by the waste management authority. Of the sample of respondents, 29.48 % stated that the waste collector collects waste from their household from 10 a.m. to 12 p.m. while 18.22 % opined about the waste collection was from 12 p.m. to 02 p.m. A large proportion (38.67 %) of the respondent reported that, they pay 50–99 taka (1US$ = 105) per month.

#### 3.7.2 Existing practice of source separation

The opinion of households on the existing practice of waste separation in the study area is presented in Table 13. The respondent households were asked about the existing practice of source separation. The interesting finding is that 67.11 % of respondents stated they separate recyclable materials sometimes while 28.44 % of respondents opined that they don't separate.

The study reveals that 78.81 % of 75 households use a bin for waste storage in their house while 16.30 % use plastic/polybags. Of the respondents, 96.30 % store their generated waste as mixed waste and 3.40 % opined that they store it as separate waste. The reason behind the source separation was investigated during the survey. The respondent expressed that, their maid collects the separated waste (Inorganic waste) from their household and sells the recyclable portion to the feriwala and uses the non-recyclable portion during cooking. Feriwala plays an important role to collect their separated recyclable material. Generally, they carry a van to the household and take the waste instead of money or food.

In the study area, there were found a large percentage (28.44 %) of people who don't separate and sell recyclable materials. The reasons behind the not willing to separate biodegradable and non-biodegradable waste were lack of awareness (26.67 % respondents) lack of sensitivity (21.78 % respondents), difficulty and time taking (22.67 % respondents), mixed waste collection system (25.19 % respondents).

#### 3.7.3 Expecting practice of waste management

The household opinion on expecting practice in waste management in the study area is presented in Table 14. The respondent households were asked about the expected practice of waste management.

According to the study, there is a willingness among residents in the surveyed area to pay the waste collector. Almost 44.74 % of respondents indicated their willingness to pay between BDT50 to BDT<100 (equivalent to 1 US$ = BDT 105) per month, while 27.26 % of households preferred to pay between BDT 20 to BDT <50. However, 23.11 % of respondents expressed disinterest in paying the waste collector. Their reason for this was that the current waste management system does not require them to pay any fees. Due to their lack of awareness, they dispose of their waste in open areas or drains and therefore do not see the need to pay.

The survey results reveal that 42.96 % family expected that, the schedule for waste collection from their house should be 12 p.m. to 02 p.m. The respondent opined that, generally there prepare their lunch meal from 12 p.m. to 02 p.m. Maximum waste generates in households during the said period due to preparing their meal. If waste collects from households after preparing their lunch meal, the maximum portion of waste will be sent. Therefore, the kitchen room will be fresh and odorless. In addition, 30.52 % of respondents in the study area expressed their opinion that the schedule for waste collection from households should be 10 a.m. to 12 p.m. By far the largest percentage (58.67 %) of the respondents opined that the preferable distance from the house to the secondary disposal point should be < 250 yd (1 yd = 0.9144 m). It is a good sign that 61.48 % of respondents have concerns about the environment. Surveys showed that a large portion of people doesn't have an awareness on waste management and their responsibility. They opined that people's motivation can be increased in waste management through seminars (40.74 % respondents) and By TV/Radio/Newspaper (24.30 % respondents). Social media such as Facebook or Instagram can play an important role to motivate the household. 34.96 % of respondents opined that Facebook and Instagram can be used as a tool to motivate people regarding waste management.

### 3.8 Proposed management process for household waste

**Fig. 16:**
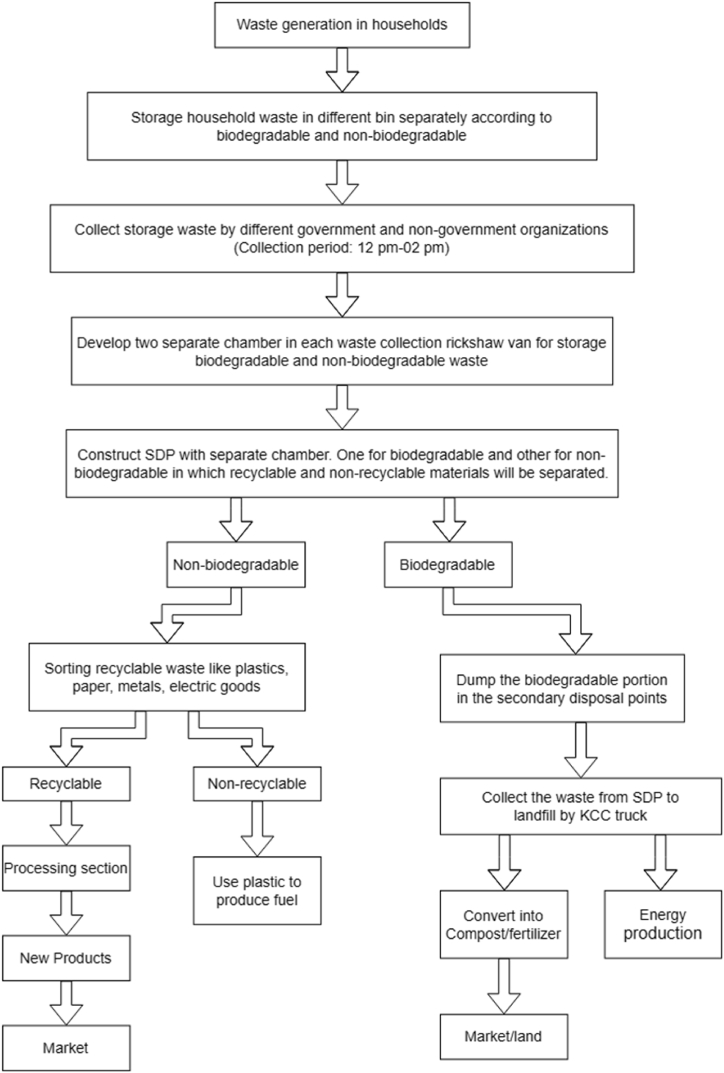
Proposed management process of household waste.

Considering the present status of municipal solid waste management in the country, the researcher has summarized in a flow chart as depicted in Fig. 16 for household solid waste management. The study shows that source separation from household solid waste plays an important role to increase the percentage of recovery of recyclable waste from mixed waste. In this proposed process, the wastes that are generated in the household will be separated into recognized types and stored in different bins according to their types. Different color bins are used for storage. It is helpful to differentiate the storage materials. In addition, proper collection of household waste is an important factor that helps to develop sustainable waste management. 42.96 % of respondents opined that the source-separated waste will be collected during the period of 12 p.m.–02 p.m. (Table 14). So the proposed waste collection schedule should be 12 p.m.–02 p.m. In the existing waste collection, rickshaw vans that are associated with the waste collection have only one chamber.

Two separate chambers in the rickshaw van for biodegradable and non-biodegradable waste will be developed and collected separated waste will be dumped at the different chambers in the rickshaw van. Sorting systems according to biodegradable and non-biodegradable waste are applied in the secondary disposal point after proper collection by local NGO or KCC. In addition, the distance between households to SDP should not exceed 250yd (1yd = 0.9144 m). Bangladesh is facing a load-shedding problem at severe now. The government is trying its best to solve the problem. Solid waste can be an alternative source of electric energy production by proper processing. Since there is a high content of compostable waste (81.62 %) in the solid waste composition of the KCC area, composting is obviously a viable option for reducing the load on land facilities. At the same time, revenue can be earned from the sale of compost as organic fertilizer. Literature shows that the moisture content of solid waste for compost should not be less than 55 %. If the moisture content is below 55 %, fungi tend to be the dominant organisms. The organic matter will not fully decompose, and good, finished compost will not be produced. Finding reveal that the moisture content of household organic waste in the study area is 74.86 % which is suitable for composting (Table 7). By proper processing, the product can be sent to the consumer's hand.

In the study area, the plastic fraction in the mixed waste was found 4.74 % (Fig. 12). This huge percentage of plastics can be used to produce new products and fuel production.

In the processing section, plastic waste will be categorized into two types namely recyclable and non-recyclable. The recyclable materials will be sent to recycle shop while the non-recycled portion will be sent to the plastic-to-fuel industry. There is a project name 3R Pilot Initiative Implementation Project which will produce fuel from plastic. The construction phase is running. The non-recyclable plastic will be used in this project to produce fuel.

## 4 Conclusions

Solid waste management strongly depends on the available waste characterizations. Different management options exist for the different fractions, due to water content, biodegradability, risk or hazardous level associated with it. Therefore, at the beginning of any waste master plan development, a comprehensive waste analysis is required. Managing the considerable amount of waste generated in various cities and towns is a challenging task, mainly due to the uncontrolled migration of people from rural areas to urban regions in pursuit of improved living standards. As a result, the production of waste in residential areas in Bangladesh is growing rapidly. The study showed the household waste generation rate is 0.472 kg per person per day. The physical composition of the waste indicates that it consists of a blend of various components, with a notable percentage (81 %) being capable of composting (biodegradable waste). The significant organic content highlights the need for regular collection and disposal, as well as the promising potential for recycling organic waste through composting. Besides, a large percentage of the waste was identified as paper (6.67 %) and plastic (4.74 %).

The moisture content and density for biodegradable and plastic were 74.86 and 16.54 % and 553.8 and 43.62 %, respectively. The result reveals that, in higher socioeconomic households, the waste generation rate was higher than the other lower-income households. The waste generation rate of households for a high-income family was 0.65kg/cap/day. On the other hand, this rate got almost half for a low-income family and its value is o.312kg/cap/day.

Source separation of waste plays a vital role to reduce plastic leakage to the secondary disposal sites. The result shows the proportion of plastic in mixed waste after sorting by the waste collector was 4.04 %. Besides, this percentage was reduced due to the separation of waste at the source. The plastic fraction was 2.99 % in the source-separated waste after sorting by the waste collector. To create comprehensive solutions for managing waste-related issues, it is necessary to involve the public in the process. Survey results show that 42.96 % of respondents opined that the source-separated waste should be collected during the period of 12 p.m. to 02 p.m. The finding suggested developing a proposed management process for household waste. Most of the households (53.93 %) in the study area were found to take collection services from the local waste management authority and were highly satisfied. The suggested model is among the most effective methods of instilling appropriate Proposed model is one of the best ways to inculcate proper solid waste management and recycling behavior in the minds of people.

## Data availability statement

Data will be made available on request.

## CRediT authorship contribution statement

**A.A. Noman:** Conceptualization, Data curation, Formal analysis, Methodology, Software, Writing - original draft, Investigation. **Islam M. Rafizul:** Conceptualization, Supervision, Validation, Visualization, Writing - review & editing. **S.M. Moniruzzaman:** Conceptualization, Supervision, Validation, Visualization, Writing - review & editing. **E. Kraft:** Funding acquisition, Supervision, Writing - review & editing. **S. Berner:** Conceptualization, Formal analysis, Investigation, Methodology, Software, Supervision, Validation, Visualization, Writing - review & editing.

## Declaration of competing interest

The authors declare that they have no known competing financial interests or personal relationships that could have appeared to influence the work reported in this paper.
